# Adapting ChatGPT for Color Blindness in Medical Education

**DOI:** 10.1007/s10439-024-03656-0

**Published:** 2024-11-27

**Authors:** Jinge Wang, Thomas C. Yu, Michael S. Kolodney, Peter L. Perrotta, Gangqing Hu

**Affiliations:** 1 Department of Microbiology, Immunology and Cell Biology, West Virginia University, Morgantown, WV 26506 USA; 2 Sona Dermatology, Bethesda, MD 20817 USA; 3 Department of Dermatology, West Virginia University, Morgantown, WV 26506 USA; 4 Department of Pathology, Anatomy, and Laboratory Medicine, West Virginia University, Morgantown, WV 26506 USA

**Keywords:** ChatGPT, GPT-4V, Color blindness, Dermatology, Pathology, Radiology, Medical education

## Abstract

Color vision deficiency (CVD) affects a significant portion of the population, yet its impact is often overlooked in medical education, especially in visually demanding specialties like dermatology, pathology, and radiology. In this study, we investigated the potential of ChatGPT to comprehend CVD-simulated images in image-based diagnostic tasks. Notably, the model successfully adapted its diagnostic reasoning to match CVD-modified color perception while preserving high prediction accuracy. These findings highlight the potential of using ChatGPT to foster more inclusive learning environments for individuals with CVD in visually intensive medical specialties.

**Supplementary Information:**

The online version contains supplementary material available at 10.1007/s10439-024-03656-0.

Color vision deficiency (CVD) affects approximately 8% of the population but remains largely overlooked in medical education, particularly in visually intensive specialties such as dermatology [1] and pathology [2]. This oversight presents significant challenges for medical students with CVD, who may struggle to interpret color-dependent diagnostic images and communicate color-based findings with colleagues and patients who perceive colors differently. Addressing this gap is essential to foster inclusivity and equity in medical education.

Traditional machine learning (ML) models have significantly advanced image-based diagnostics. However, they require retraining to adapt to new tasks and often function as "black boxes," without offering insight into their decision-making processes. In contrast, Generative Pre-trained Transformers with visual capabilities, such as ChatGPT-4Vision (hereafter denoted as GPT-4V), can adapt to new tasks through few-shot learning without altering their training parameters and engage in dialogues about findings from images. These features led us to hypothesize that adapting GPT-4V to the altered color perceptions of CVD could serve as an innovative assistive tool, accommodating the unique needs of individuals with CVD in medical education.

As a proof of concept, we designed a few-shot learning framework that adapts GPT-4V for specific types of CVD and demonstrated its application in a fundamental task in medical education: classification of skin lesion images obtained from dermoscopy (Fig. 1a). Few-shot learning allows models to adapt to new context (CVD in our case) using only a small number of reference images [3–5]. The key innovation in our approach is the simulation of both query and reference images to match the same CVD condition. Taking protanopia (red deficiency) as an example, for each image with its original color, we employed established methods [6] to convert it into an image reflecting how individuals with protanopia perceive the image (see examples in Fig. 1b). This converted image was sent to GPT-4V for classification using a few-shot learning framework, where reference images were similarly simulated under the same CVD condition. The same procedures were applied to deuteranopia (green deficiency) and tritanopia (blue deficiency).

**Fig. 1:**
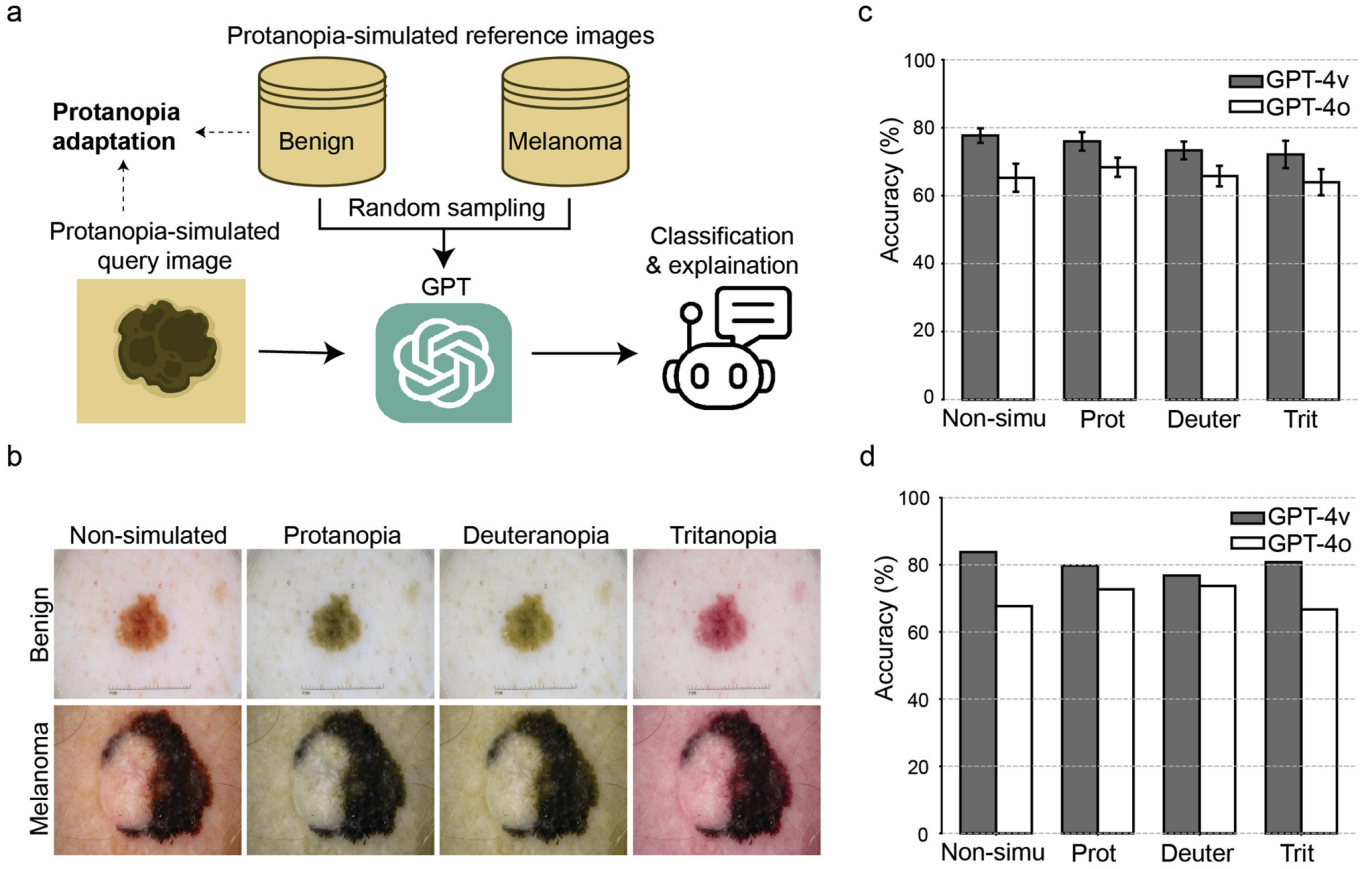
Adaptation of GPT-4V to CVD and application to dermoscopic classification. **a** Flowchart of adapting GPT-4V to CVD through few-shot learning with an application to image classification, shown protanopia as an example. For each CVD-simulated query image, few-shot learning randomly selected reference images (also simulated to the same condition) from each category for classification purpose. **b** Examples of non-simulated and CVD-simulated dermoscopic images. The top row shows images of a benign nevus under non-simulated, protanopia, deuteranopia, and tritanopia conditions. The bottom row shows images of a melanoma diagnosis. Non-simulated color images were sourced from the International Skin Imaging Collaboration archive (https://www.isic-archive.com/): ISIC_0046725 for a melanoma and ISIC_0012656 for a benign nevus. **c** Average classification accuracies of GPT-4V for non-simulated and CVD-simulated images. Error bars: standard deviations. All experiments were in ten repeats. **d** Classification accuracy of GPT-4V following the application of the consensus strategy. GPT-4o (version “gpt-4o-2024-05-13”) included for comparison. Abbreviations: Non-simu (non-simulated images), Prot (protanopia-simulated), Deuter (deuteranopia-simulated), and Trit (tritanopia-simulated).

Our first objective was to assess whether adapting GPT-4V to CVD-associated color perception would affect its prediction accuracy. We tested the framework using a balanced set of 100 histopathology-validated dermoscopic images [7, 8]. For each query image, we followed established few-shot learning protocols [5] to randomly sample examples from the remaining images, identify the most similar image, and use its label for prediction. Since model accuracy stabilizes after one example, we sampled two examples for each category and repeated the experiment ten times. We prompted GPT-4V through Application Programming Interface (version “GPT-4-Turbo-2024-04-09”) for this adaptation task.

GPT-4V achieved accuracies of 76.0% ± 2.7% for protanopia, 73.3% ± 2.6% for deuteranopia, and 72.1% ± 4.0% for tritanopia simulations, closely comparable to the 77.7% ± 2.1% accuracy for non-simulated images (Fig. 1c). Implementing a consensus strategy—classifying an image as “melanoma” if identified as such in at least five out of ten repeats—further improved accuracy to 79.8% for protanopia, 76.8% for deuteranopia, and 80.8% for tritanopia simulations, approaching the 83.8% accuracy for non-simulated images (Fig. 1d). For comparison, a previous study found that vision-normal medical students achieve accuracies of 78.1%, 65.4%, and 74.2% for these respective simulations [9]. These results demonstrate that GPT-4V’s adaption to the altered color perception of CVD did not significantly compromise its diagnostic performance.

An ablation test underscored the importance of matching the CVD conditions of the reference images to those of the query images. In this test, we fixed the CVD simulation for the query images and compared scenarios where reference images were either simulated to match or left non-simulated. Accuracy was 4–8% higher when the CVD conditions of the query and reference images matched, particularly for protanopia and deuteranopia (Fig. 2a). This emphasizes the necessity of consistent simulation in both query and reference images to optimize the model's performance.

**Fig. 2:**
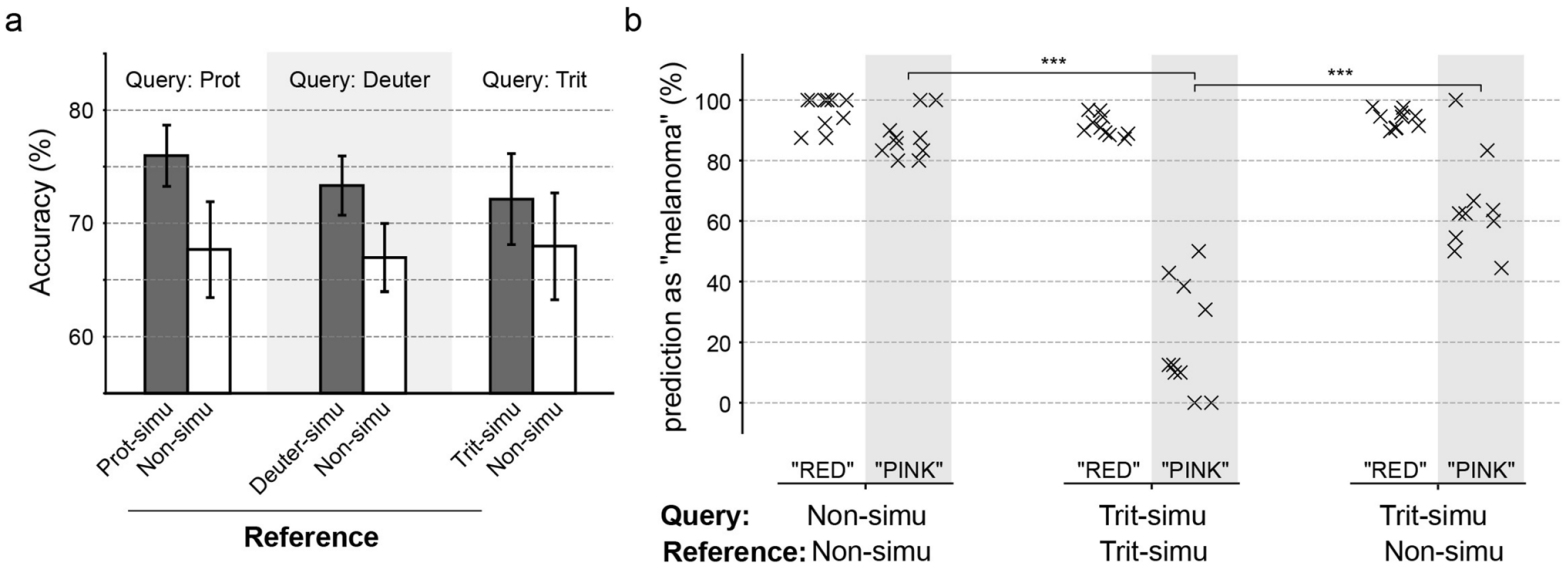
Ablation tests validating the importance of matched CVD simulation between query and reference images. **a** Accuracy comparison where simulation of a CVD condition was fixed for query images, and reference images were either matched in simulation (gray bars) or left non-simulated (white bars). **b** Comparative analysis of the association between mentioning red/pink color in explanations and “melanoma” prediction in non-simulated and tritanopia-simulated images. Two groups of image inquiries were identified based on GPT-4V’s explanations on its predictions. The first group, labeled “RED,” included explanations where red color is mentioned to describe query image, regardless of the pink color. The second group, labeled “PINK,” comprised explanations mentioning pink without red. The Y-axis displays the percentage of “melanoma” predictions from each replicate. ***p < 0.001 (*t*-test; two-tail).

A key advantage of GPT-4V over traditional ML models is its ability to provide textual explanations for each prediction. An intriguing finding from our analysis of these explanations was how GPT-4V interpreted pink or red colors. Under non-simulated conditions, the model identified these colors as indicative of melanoma (Fig. 2b; left two columns). However, when images were simulated for tritanopia—where pink hues become more prevalent—the model's classification criteria shifted, associating pink coloration with benign nevi (Fig. 2b; fourth column). In ablation test where non-simulated reference images were used with tritanopia-simulated queries, pink was again associated with melanoma (Fig. 2b; sixth column). Detailed numbers of true positives, false positives, true negatives, and false negatives further supporting the observations were shown in Supplementary Table 1. These findings demonstrate GPT-4V's ability to adapt its reasoning and explanation based only on a few numbers of examples with matched color perceptions—a capability not available in traditional AI models.

The limitations of this study include its demonstration on a classification task of dermoscopic images with a relatively small sample size. Nevertheless, our study provides a proof of concept that few-shot learning can align GPT-4V's diagnostic rationale with the altered color perception of CVD while maintaining high diagnostic accuracy. Future research involving medical students with CVD is essential to further assess the effectiveness of GPT-4V in overcoming color perception challenges. Additionally, expanding this evaluation to other image-intensive fields, such as pathology and radiology, could broaden its impact and contribute to more inclusive medical education.

## Supplementary Information

Below is the link to the electronic supplementary material.

Supplementary file1 (PDF 186 KB)

## Data Availability

Code for color blindness simulation and copies of GPT-4V’s responses are accessible at GitHub: https://github.com/JingeW/CVD-Dermoscopic-Image-ICL-GPT4.
