# Supplementary material for: Adapting ChatGPT for Color Blindness in Medical Education
Source: Ann Biomed Eng. 2024 Nov 27;53(1):5–8. doi: 10.1007/s10439-024-03656-0 (PMC11782439; doi:10.1007/s10439-024-03656-0)
Supplement: Supplementary file 1 — Supplementary file1 (PDF 186 KB) [file 10439_2024_3656_MOESM1_ESM.pdf]

Supplementary Table 1: Details of TP, FP, TN, and FN by query-reference configuration and presence of "red" or "pink" in diagnosis explanation

Query	Reference	Sorted by key words in "Explaination"	TP	FP	TN	FN	% predicted as "melanoma"
Non-simu	Non-simu	"RED": red regarless of pink	116	11	0	6	95.5%
Non-simu	Non-simu	"PINK": pink only	62	1	1	6	90.0%
Trit-simu	Trit-simu	"RED": red regarless of pink	199	64	11	13	91.6%
Trit-simu	Trit-simu	"PINK": pink only	15	3	42	46	17.0%
Trit-simu	Non-simu	"RED": red regarless of pink	225	150	12	2	96.4%
Trit-simu	Non-simu	"PINK": pink only	35	16	15	17	61.4%

*The numbers are aggregated from 10 replicates.
